# REPRESENT recommendations: improving inclusion and trust in cancer early detection research

**DOI:** 10.1038/s41416-023-02414-8

**Published:** 2023-09-09

**Authors:** Frederike Brockhoven, Maya Raphael, Jessica Currier, Christina Jäderholm, Perveez Mody, Jackilen Shannon, Bella Starling, Hannah Turner-Uaandja, Nora Pashayan, Ignacia Arteaga

**Affiliations:** 1 Department of Applied Health Research, University College London, London, UK; 2 Department of Social Anthropology, University of Cambridge, Cambridge, UK; 3 Division of Oncological Sciences, Oregon Health & Science University, Portland, OR USA; 4 School of Public Health, Oregon Health & Science University—Portland State University, Portland, OR USA; 5 Vocal, Manchester University NHS Foundation Trust, Manchester, UK; 6 Early Cancer Institute, University of Cambridge, Cambridge, UK

**Keywords:** Social anthropology, Research management, Ethics

## Abstract

Detecting cancer early is essential to improving cancer outcomes. Minoritized groups remain underrepresented in early detection cancer research, which means that findings and interventions are not generalisable across the population, thus exacerbating disparities in cancer outcomes. In light of these challenges, this paper sets out twelve recommendations to build relations of trust and include minoritized groups in ED cancer research. The Recommendations were formulated by a range of stakeholders at the 2022 REPRESENT consensus-building workshop and are based on empirical data, including a systematic literature review and two ethnographic case studies in the US and the UK. The recommendations focus on: Long-term relationships that build trust; Sharing available resources; Inclusive and accessible communication; Harnessing community expertise; Unique risks and benefits; Compensation and support; Representative samples; Demographic data; Post-research support; Sharing results; Research training; Diversifying research teams. For each recommendation, the paper outlines the rationale, specifications for how different stakeholders may implement it, and advice for best practices. Instead of isolated recruitment, public involvement and engagement activities, the recommendations here aim to advance mutually beneficial and trusting relationships between researchers and research participants embedded in ED cancer research institutions.

## Introduction

Cancer is a leading cause of death worldwide, accounting for almost ten million deaths in 2020 [1]. Policymakers and experts hold that one of the most effective ways to improve cancer outcomes is by detecting it early [2]. Yet those negatively affected by power and privilege based on a characteristic they share (such as income, race, ethnicity, sexual identity, geographic location or disability), henceforth referred to as minoritized groups^1^, face multiple barriers to participating in cancer research [4, 5]. As a result, they are underrepresented in early detection (ED) cancer research despite being disproportionately affected by cancer [6]. This underrepresentation leads to critical gaps which compromise the generalisability of study findings and exacerbate disparities in cancer outcomes for members of minoritized groups [7].

While inclusive cancer research with minoritized groups has its challenges, the nature of ‘early detection’ poses further challenges. In contrast to late-stage cancer research, those sought as participants for ED cancer studies are usually cancer free or asymptomatic. Hence, the potential benefits of participation, such as access to experimental and otherwise unavailable treatments, taking ownership of one’s health and increased knowledge of a condition, are less obvious in ED cancer studies. Furthermore, participation in ED cancer research poses additional risks such as false positive results and overdiagnosis that may require invasive and potentially unnecessary follow-up treatment (*see* Table 1*: Definitions of terms used in this manuscript*). These factors influence the stakes of participation in cancer research for members of minoritized groups who are more likely to be uninsured or lack access to follow-up care [8–10].

**Table 1:** Definitions of terms used in this manuscript.

Term	Definition
Cancer early detection (ED)	Finding and diagnosing cancer in its earliest stages, either before the disease becomes symptomatic or quickly after symptoms start showing, so that the cancer can be treated before it has a chance to spread.
Community Champions	Selected members of a community who have received training to educate other members of their community about certain (health and research related) topics. Also referred to as peer educators, community lay health workers, and community (health) educators.
Community Engagement Organisation	An organisation specialised in liaising and collaborating with members of a particular (minoritized) group or groups. These organisations function as gatekeepers or connectors between outside organisations and members of those groups, understanding the languages, goals and concerns of both sides.
Community practitioners	People who are embedded within communities and have specific skills, knowledge and experience related to the issues affecting these by virtue of their membership to the group.
Cultural humility	Stemming from the criticism that ‘cultural competence’ suggests that there is an endpoint to becoming culturally competent and that a group of people can be understood by a (single) shared characteristic, cultural humility promotes a lifelong process of self-reflection, acknowledging one’s own identities and biases, and understanding that each human being has a complex identity of (intersectional) characteristics [37].
Engagement practitioners	Professionals who are specialists in involvement and engagement who work within the overall environment of health research. Their role is to coordinate, design, manage and facilitate public engagement in health research. Moreover, they often act as connectors between researchers and communities and can be ‘guardians’ of trust within research relationships.
False negative result	A test result indicates a given condition does not exist when it does.
False positive result	A test results indicates a given condition exists when it does not.
Inclusion	An effort is made to ensure that certain groups are not excluded based on a shared characteristic (e.g., their religion, income or abilities).
Institutions	In the context of this paper, with institutions we mean universities, teaching hospitals, or any other establishment that employs and oversees researchers.
Minoritized groups	Those negatively affected by power and privilege based on a characteristic they share (such as income, race, ethnicity, sexual identity, or disability).
Overdiagnosis	Detection of cancer through screening that otherwise would not have been noticed during a person’s lifetime.
PPIE	An active partnership between members of the public and researchers that influences and shapes research [117].
Public contributors	Members of the public who contribute to a conversation or (research) process by sharing their view as a layperson.
Public Involvement	‘Research being carried out ‘with’ or ‘by’ members of the public rather than ‘to’, ‘about’ or ‘for’ them.’ This means that members of the public participate in the decision-making, planning, implementation and evaluation of research studies on topics that affect them [117].

Extensive research has focused on the direct and indirect barriers minoritized groups face to participate in medical research [4, 5, 11–17]. For example; narrow inclusion/exclusion trial eligibility criteria (e.g. age, pregnancy, lack of other medical conditions) [18], inaccessible promotion and communication of trial materials (e.g. provision of written information in only one language) [12, 18, 19], negative financial impact [19], cultural barriers [11, 19], participants’ perception of risk or ability to influence the research [12, 19], fear of not being treated with dignity and respect, and mistrust of the healthcare system [11]. Indeed, empirical data has shown trust to be one of the most crucial factors impacting participation in biomedical research [20]. Yet building and maintaining trust takes time, often beyond the scope and timelines of clinical trials [21]. This paper therefore situates the researcher/participant relationship as embedded within the wider ED cancer research infrastructure, and attends to issues of trust and access that arise from particular national, cultural and social contexts (with a focus on the US and the UK).

Against the backdrop of these unique challenges, the study ‘REPRESENT: A Community Engagement Roadmap to Improve Participant Representation in Cancer Research Early Detection’ set out to advance practical steps to build relations of trust and include minoritized groups in ED cancer research. Based on a range of methods including a systematic review of published literature and two ethnographic case studies in the United States (US) and the United Kingdom (UK) (see appendix); this paper presents twelve recommendations (see Table 2). These were formulated at the 2022 REPRESENT consensus-building workshop and emerged through two questions co-created with diverse stakeholders^2^: How can all communities be included in cancer early detection research? How can trust be built between cancer early detection researchers and underrepresented groups?

**Table 2:** Twelve recommendations to include everyone in cancer ED research and build trust between researchers and minoritized communities.

#1	**Establish long-term connections and trusting relationships with minoritized groups (not bound by specific research projects and funding)**.
	a. Funders allocate multi-year budgets to institutions to establish long-term programmes between researchers and members of minoritized groups/community organisations.
	b. Institutions allocate budget and resources to long-term permanent programmes between researchers, community engagement practitioners and members of minoritized groups/community organisations.
	c. Research teams work collaboratively with both community and engagement practitioners to establish trusting, long-term relationships with members of minoritized groups and local community organisations through open-minded conversations.
	**Advice for Good Practice:**
	• Enable ‘community sensitisation’, the mutual exchange of information about research and community characteristics before any research-related activities happen^a^.
	• Work with community engagement practitioners and community liaisons to help shift the institutional power dynamics [118]^a^.
	• Include people from minoritized groups in research protocol design and grant review panels at institutions [119].
	• Work with local community organisations to understand groups’ priorities and concerns beyond the concrete research topic (e.g. through town halls, discussion groups, informal discussions, online communications)^a^.
	• Share available resources in the institution with groups (see recommendation #2).
	• Train members of minoritized communities to become peer educators (see recommendation #4).
	• Representatives from research teams/ institutions attend community-led events (e.g. local/cultural celebration, health fair, rally, or religious celebration, when appropriate and invited)^a,b^.
#2	**Establish systems and processes to share resources and expertise with minoritized groups to help address some of their needs and priorities**.
	a. Funders create/allocate funds to community-led research grants to be administered in partnership with existing research institutions.
	b. Institutions create ‘community-led research programmes where minoritized groups/community organisations can apply for funding to address their own research priorities by collaborating with researchers and accessing the institution’s resources (See: [120, 121]). As well as community engagement posts to signpost resources and support research teams in liaising with communities.
	c. Engagement practitioners collaborating with research institutions map resources and expertise already available in the institution, and share opportunities with minoritized groups.
	**Advice for Good Practice:**
	• Organising educational or Q&A sessions about topics chosen by the community [122].
	• Sharing useful medical knowledge in dedicated sessions^a^.
	• Helping with university applications or securing internships in related fields for members of minoritized communities as part of outreach efforts.
	• Lending support in grant applications, project design, or legal proceedings [123].
#3	**Ensure all study materials are culturally sensitive, translated into appropriate languages, and accessible in their format, language and dissemination**.
	a. Funders allocate sufficient budget and time for research teams to prioritise culturally sensitive and accessible design, translation and dissemination of all study materials.
	b. Institutions make services such as interpretation, translation and graphic design available to research teams.
	c. Research teams work with representatives from minoritized backgrounds/engagement practitioners to develop culturally sensitive and accessible design and dissemination of all study materials.
	**Advice for Good Practice:**
	• Prioritise face-to-face interaction about the research with members of minoritized groups where possible to foster and maintain trust [6, 37, 119, 124–126].
	• Disseminate study materials in multiple and accessible formats (e.g. video, email, text, phone, in person, social media), languages (incl. low literacy English) and occasions (before, after and during the study) [37, 127]^a^.
	• Use simple and clear language (plain English/lay language), avoid the use of scientific jargon and complex terminology, provide relevant information in the right order, and ensure the design is legible (font size, formatting, colours) [127].
	• Ensure that translation and interpretation services maintain the tone, level and relevance of the original text [100, 127].
	• Work collaboratively with engagement practitioners, multicultural agencies and community organisations to create messaging and patient information sheets that are inclusive and accessible in tone and content^a^.
	• Display cultural sensitivity, respect, and awareness of potential cultural, generational and linguistic barriers that minoritized groups may face in communication design and dissemination [128].
	• Ensure the transparency, consistency, use of credible sources/data and display official logos of institutions in research-related communications to avoid suspicion and distrust [129]^a^.
#4	**Train and recruit community champions to become peer educators on cancer ED and help promote study participation**.
	a. Funders allocate budgets for peer educator recruitment and training.
	b. Institutions lead peer educator programmes by creating and updating databases of peer educators and providing (guidance on) training.
	c. Research teams or the institutions’ engagement practitioners recruit peer educators, provide training for particular research project needs and foster mutually beneficial relationships with them.
	**Advice for Good Practice:**
	Transferable skills and access to opportunities that can be included in peer educator training:
	- Knowledge of cancer, cancer early detection, and cancer research
	- Leadership and public speaking skills
	- Best practices in teaching
	- Best practices in research recruitment
	- Research ethics including the risks and benefits of participating in clinical research
	- Communication, messaging and social media skills
	- Experience organising (community) events
	- Access to career, apprenticeship and internship opportunities in cancer ED networks
	- Providing mentors in the field of cancer ED
#5	**Transparently and accessibly communicate the benefits, risks and expectations of participating in cancer ED studies to potential participants, including the possibility of overdiagnosis and overtreatment**.
	a. Funders ensure research proposals minimise risks for participants, and require research teams to make detailed arrangements for follow-up care.
	b. Research teams make a plan (with input from participants) on how and when to communicate risks and benefits to participants in an accessible and timely manner.
	**Advice for Good Practice**
	• Ensure patients can ask questions and express concern in an accessible way (see recommendation #3) [44].
	• Consider the setting in which these conversations take place; a hospital/clinical setting may arouse negative associations and mistrust^a^.
	• Be transparent from the outset of the study (enrolment stage) that there is a likelihood that participants’ results may be uninformative or uncertain from a diagnostic perspective [46].
	• Ask for guidance from community organisations to ensure that communications, including informed consent processes, and spaces in which they occur are accessible and trustworthy for the groups you are trying to reach^a^.
#6	**Grant minoritized groups appropriate compensation and support for participating in cancer ED research**.
	a. Funders allocate a budget for appropriate compensation and incentives.
	b. Institutions develop clear and accessible processes that allow for flexible and diverse methods of compensation for participation.
	c. Research teams determine, in collaboration with representatives from minoritized groups, which compensation and incentives would be useful/appropriate and deliver them.
	**Advice for Good Practice:**
	• Co-develop a clear policy on payment and compensation for participation with contributors from minoritized groups/community organisations to ensure no important barriers are missed, and that payment methods are appropriate and communicate it to participants before enrolment.
	• When deciding on compensation for research participation, look into additional costs for participants: missed work, childcare and family duties, transportation, and the cost of a carer/translator/companion all add to the opportunity cost of participation.
	• Provide additional support in cases of research that is conducted online/via phone such as paying for data, WIFI, or minutes and/or securing a private, quiet space.
	• Investigate how recipients of benefits or other state financial support are affected by the proposed compensation [130].
	• Communicate clearly what compensation covers (e.g., participation in clearly outlined research activities) and what it does not (e.g., health insurance, treatment) so participants can make an informed choice.
#7	**Use representative samples in cancer ED trials and document implemented and evaluated engagement/recruitment approaches. If a study does not use a representative sample, it must explain/justify why this is the case**.
	a. Funders make representative and inclusive sampling a primary consideration when reviewing grant applications, requiring researchers not using representative samples to justify why this is the case. Additionally, funders should mandate in grant applications that researchers document attempted engagement and recruitment approaches.
	b. Institutions make engagement and recruitment approaches used in research publicly available.
	c. Research teams use the four Trial Forge questions to ensure their samples are representative [21]. Document implemented and evaluated engagement and recruitment approaches and share this information with the institutions, funders and/or in published articles.
	d. Academic journals and publishing outlets encourage authors to publish recruitment and engagement approaches as part of the methodology when submitting manuscripts.
	**Advice for Good Practice:**
	• Use Trial Forge questions at the outset of the research design [21].
	Documentation of recruitment and engagement approaches should include:
	- Diversity within the research team and people tasked with recruitment/engagement.
	- Locations of recruitment/engagement.
	- Duration of recruitment/engagement.
	- Methods of recruitment and engagement (e.g. partnership with community organisations, health fairs, social media posts) perceived benefits and limitations of these methods, and inclusivity considerations.
	- Methods for retention of participants (including retention rates).
	- Incentives, compensation and resources offered to participants (e.g., transportation, interpreting, payment).
	• Use Trial Forge questions at the outset of the research design [21].
#8	**Collect, analyse and share data on participant demographics in cancer ED studies**.
	a. Funders incentivize the collection of aggregate data on research participants’ characteristics in grant applications.
	b. Institutions train researchers to responsibly collect, store and share aggregate data on relevant participant characteristics.
	c. Research teams collect and share data on the characteristics of study participants, in accordance with data protection laws.
	d. Academic journals and publishing outlets require researchers to include data on relevant characteristics of research participants when submitting manuscripts.
	**Advice for Good Practice**
	• Transparently explain to participants why these data are collected and how they will be handled under safeguards and data protection regulations (aggregation, anonymization, restricted access to data storage).
	• Give participants the option to answer in a multiple-choice format, where possible, so that they feel less exposed.
	• Participants should always have and be made aware of the option not to answer questions.
	Recording the following characteristics when conducting a study has already been suggested as standard practice [119]:
	- The disease, problem, or condition under investigation
	- Special considerations related to sex and gender, age, ethnic group, and geography.
	- The overall representativeness of the trial, including how well the study population aligns with the target population in which the results are intended to generalise.
	Workshop participants suggested also adding:
	- Sexual orientation
	- Gender identity [90]
	- Disabilities
	- Religious affiliation
	- Collecting data on postcodes rather than broader ‘geography’, when relevant.
#9	**Create an appropriate communication and support plan for participants for whom the cancer ED study detects increased risk of cancer or lesions**.
	a. Funders request that a detailed plan for such a scenario is outlined in grant applications and ensure that proposed follow-up care is covered by appropriate institutions.
	b. Institutions support research teams by signposting resources, services, and best practices.
	c. Research teams co-produce a detailed plan of whether/how/when to communicate findings to affected participants, and how further testing, referrals and counselling will be arranged.
	**Advice for Good Practice**
	• Co-produce a follow-up plan with input from relevant public contributors.
	• Employ a non-clinical participant navigator on the research team to support individuals with needs relating to a positive result.
	• Budget for community specific, accessible dissemination including but not limited to translations and interpretation for live events.
	• Provide ways for participants to reach out throughout the research cycle.
#10	**Disseminate study updates and results in an accessible and timely manner, expressing gratitude to participants for their contribution**.
	a. Funders allocate a budget for research teams to share updates and results with participants, considering that the deadline to use this budget could extend beyond the end of the grant.
	b. Research teams plan and budget to give participants regular updates on the research in appropriate formats and languages.
	**Advice for Good Practice:**
	• Clearly and accessibly communicate to participants exactly when and how results will be shared with them from the outset of the study (For example see Healthy Oregon Project [131]).
	• Disseminate results via multiple formats and channels [132].
	• Consider providing the following content: factual information, health implications and general research information [83].
	• Relay results to participants as soon as possible (fieldwork participants expressed their desire for results in 6–10 weeks). In cases when results will take a long time, explain this to participants from the outset and offer sessions where they can ask questions and express concerns in the meantime^a^.
	• Express gratitude to participants and demonstrate that their time, efforts and contribution are valued [127, 133].
#11	**Mandate training on inclusive community engagement approaches with minoritized groups**.
	a. Funders mandate training through grant applications, provide resources for high-quality training, and signpost available training to researchers whose institutions do not yet provide adequate training opportunities.
	b. Institutions mandate and organise training from early stages in researchers’ careers, and signpost researchers to training if they do not provide this.
	c. Research teams attend training, ideally before applying for funding and designing a study.
	**Advice for Good Practice**
	• Develop training sessions in collaboration with appropriate community organisations [21].
	• Provide researchers with training early on in their careers (doctoral, post-doctoral, early-career).
	Topics that could be covered in training include:
	- Health disparities for minoritized groups
	- Best practices in community engagement approaches
	- Responding to (historical) experiences of marginalisation, trauma, ableism and racism in research
	- Awareness of diversity, inclusion, and equity in research
	- Developing cultural competence and cultural humility
#12	**Create inclusive employment opportunities and progression pathways in Cancer ED research for members of minoritized groups**.
	a. Funders collect equity and inclusion data beyond lead applicants and incorporate the diversity of research teams into scoring criteria when reviewing grant applications.
	b. Institutions offer inclusive recruitment, employment and progression opportunities in Cancer ED research, incentivise diversity of research teams and ensure an inclusive working culture.
	c. Research teams create inclusive hiring criteria and role descriptions and remove barriers unfairly impacting minoritized groups from the recruitment process.
	**Advice for Good Practice**
	• Create apprenticeships and internships on ED cancer research teams for members of minoritized groups (see Cancer Research UK, 2021 [97]).
	• Identify and remove barriers that minoritized researchers in cancer research face; e.g. create flexible employment policies, equitable access to financial resources and progression opportunities [97].
	• Actively attempt to increase grant, research and job applications from minoritized applicants (e.g. create grants for early career researchers as opposed to senior researchers, advertise job opportunities using inclusive communication methods, reward diverse research teams, and provide pre-application guidance and support).
	• Devise actionable Equality Diversity and Inclusion employment plans which include proposed actions, intended outcomes, timelines and metrics (See: Wellcome [134] Cancer Research UK, 2021 [97]), and conduct external/internal evaluations to monitor progress (See: The Social Investment Consultancy and The Better Org, 2022 [135]).
	• Create inclusive, transparent and equitable hiring criteria and remove requirements that disproportionately rule out applications from members of minoritized groups
	• Adopt anti-racist principles, toolkits and training, with a zero-tolerance policy for racism, discrimination and bullying [135].

^a^Informed by fieldwork.

^b^Informed by literature review.

## Methodology

### Research activities

Two ethically approved case studies, one in Oregon (US) and one in Manchester (UK), and a scoping literature review informed discussions and provided empirical evidence for the recommendations formulated at the workshops. Fieldwork in Oregon was conducted from March to April 2022 with the Community Outreach, Research and Engagement (CORE) team at the Oregon Health & Science University (OHSU) in Oregon, US. CORE carried out evidence-based research engagement models with Latino and Hispanic participants in Spanish: Two Community Engagement Studios (CES), and one using the Community Readiness Assessment Model (CRAM) [22]. Fieldwork in Manchester was carried out with Vocal, a non-profit organisation hosted by the Manchester University NHS Foundation Trust for four weeks in May 2022. Vocal carried out four community workshops with different minoritized groups in English with interpretation and translation to relevant languages when needed. Research questions in both field sites were centred on trust and participation in cancer ED research (see Appendix: Methods). The systematic scoping review identified 38 studies published between 2004 and 2022 on implemented approaches to improve participation of minoritized groups in ED cancer research.

#### Consensus-building workshop

The REPRESENT team organised a three-day virtual workshop in June 2022, with participants from the UK and US (*n* = 33) to share research findings and discuss practical recommendations to improve inclusion and trust-building with minoritized groups in ED cancer research. Four relevant stakeholder groups were invited in addition to the REPRESENT team members (*n* = 9): community representatives and public contributors who were representatives of non-governmental organisations or underserved groups (*n* = 7), researchers (*n* = 13), research managers and coordinators (*n* = 3), and one representative from a cancer research funding body. With the consent of participants, conversations during the workshop were recorded and transcribed.

**Fig. 1:**
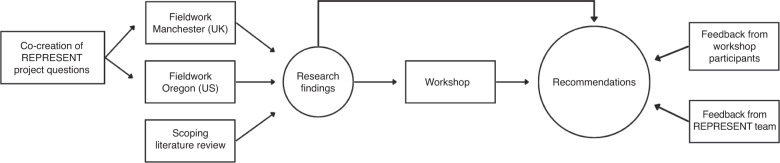
Overview of the phases to generate the REPRESENT recommendations. Research findings from case studies and literature review were discussed at a 3-day virtual workshop and translated into twelve recommendations. These were later improved via electronic correspondence with workshop participants and the research team.

The workshop was structured as follows: The first day centred on the REPRESENT teams’ research objectives and results arising from the research activities outlined above. Each presentation included an overview of relevant findings, dilemmas arising from fieldwork and time for discussion with workshop participants. On the second day of the workshop, participants were divided into breakout sessions to tackle four key questions arising from fieldwork and the literature review as reported on day one (see Table 3). On day three, a list of thirty preliminary recommendations based on relevant workshop findings and discussions was compiled by the workshop steering committee and presented to attendees for feedback. Participants were split into two breakout rooms where each group discussed half of the proposed recommendations. Recommendations focused on areas such as pre-research relationships, sharing benefits of ED research, institutionalising inclusion, and inclusive communication. Discussions were facilitated by members of the REPRESENT team and were informed by questions such as “is this recommendation feasible/clear/important/complete?”. Answers were presented to the plenary by a group delegate, followed by a discussion with the wider group to achieve consensus and address any disagreements among stakeholders. Agreed in principle during the workshop, each recommendation had to be carefully rephrased to include the nuance of the discussion. Therefore workshop participants were invited to contribute to the development of recommendations after the event through electronic correspondence with the REPRESENT team (see Fig. 1). The process to reach consensus ensured that recommendations accurately reflected the wide array of perspectives, experiences and values that each participant brought to the workshop.

**Table 3:** Questions discussed on Day 2 of the REPRESENT Workshop.

Q1.	How can we establish a pre-research relationship with members of minoritized groups? Discuss what practical steps would be taken to establish such relations
Q2.	How can we build and repair trust between researcher teams and members of community groups? Please discuss what practical steps would be taken in the context of early detection cancer research.
Q3.	What practical steps could be taken to overcome contextual privacy barriers in early detection cancer research?
Q4.	Is recruitment for research in cancer early detection different from recruitment for other types of studies?

## Recommendations

Twelve recommendations were created by the REPRESENT team and further developed with workshop participants. Table 2 sets out specifications for various stakeholders (funders, institutions, research teams, engagement practitioners, research journals) to implement them, considering their viable scope of action. Advice for best practices for each recommendation is also included in Table 2. Below, we outline the reasoning underpinning each recommendation.

### Long-term relationships that build trust

There was consensus amongst workshop participants, further reinforced by findings from the ethnographic fieldwork and systematic literature review, that building trust between researchers and minoritized groups takes a significant period of time [20, 21, 23, 24]. This presents a major challenge for research teams working on short-term projects and grants. Likewise, time commitment may pose a challenge for members of minoritized groups facing multiple daily struggles that often leave them unable to engage in long-term planning [25]. To overcome this, research partnerships between research teams and minoritized groups need to transcend the temporary nature of specific grants and projects. Moreover, if institutions and researchers want to establish trusting relations with minoritized groups they cannot do so solely on their terms and timelines [26]. This requires a rethinking of agenda-setting practices and funding restrictions that currently inform standard practices in institutions, from short-term researcher-driven projects to long-term programmes centred on community-led themes [26–30]. Organisations specialised in community engagement, either those in the community or within academic research institutions, could play a critical role in helping develop and transform relationships between research teams and minoritized groups by mediating interests and acting as guardians of trust [31, 32]. Workshop participants agreed that continuous relationships that are not transactional but mutually beneficial for all involved, help foster trust between minoritized groups, research teams and institutions. In the long-term, workshop participants suggested this would enable a culture change in the way that research is done; transforming hierarchical researcher/participant relations with communities to relations that are reciprocal and relevant to the needs of minoritized groups.

### Sharing available resources

Cancer ED research may not directly benefit participants as tests take years to move from bench to bedside and are not always successful. Often, research teams do not know whether and for whom technologies would work, which is why the study is set up in the first place. Involving everyone in research to find out whether a technology is beneficial, rather than excluding some people from the outset, will strengthen the validity and specificity of research findings [33]. However, minoritized groups may not consider cancer ED a priority among a plethora of other needs [22, 25]. To avoid developing extractive relationships with minoritized communities, where time, effort and resources are required from them without adequate compensation or acknowledgement (see recommendation #6), research teams must make an effort to incorporate activities that benefit the groups they are working with. Instead of trying to convince people why cancer ED research should be a priority in their lives, researchers might gain more insight by asking communities what they struggle with and actively seeing if their team and institutions can help address those needs [24, 34, 35]. Research teams and the institutions they are affiliated with that have access to resources and networks could be of help to communities. Sharing these is likely to strengthen trust in the relationship while making research mutually beneficial [20].

### Inclusive and accessible communication

The wider literature on (under)representation in research, the fieldwork and systematic literature review all highlighted the significance of inclusive and accessible communication in fostering trust and improving the participation of minoritized communities in ED cancer research [21, 36, 37]. When researchers do not adequately attend to the cultural, physiological and linguistic differences among minoritized groups, this can result in ineffective communication of research materials and outputs, further hindering the inclusion of these groups in research [4]. For this reason, workshop participants deemed it crucial that researchers engage in community sensitisation, which involves the mutual exchange of information about research and community characteristics, before any research related activities happen (see recommendation #1) and working with representatives from minoritized groups, engagement practitioners and/or Equality Diversity and Inclusion consultancy organisations (see Table 1) to tailor the design, content, translation and dissemination of the study materials to the diverse groups they seek to enrol. This should include not only appropriate wording of every public-facing document, but also choosing appropriate formats and mediums to cater for people with different abilities, including but not limited to those potential participants with sight or hearing impairments.

### Harnessing community expertise

One approach that has proved effective in improving recruitment and education of minoritized communities in ED cancer research is the use of community champions (see Table 1) [38–41]. Community champions are active members of a particular community who have a strong interest or lived experience of cancer and/or cancer research [41]. Fieldwork in Oregon and Manchester demonstrated that community champions can use existing relationships and lived experiences to share information, reach more people in minoritized groups, answer questions accessibly, and acknowledge and respect the norms of that group. Community champions can help create buy-in and foster trust, improving recruitment and retention rates of minoritized groups in cancer ED studies [38–40]. Research teams and institutions that design peer educator programmes can make research a mutually beneficial endeavour by teaching community champions transferable skills that serve them beyond specific research projects, in addition to compensating them appropriately for their work. In this way, peer educator models can improve both research participation in ED studies as well as transferable knowledge and skills available to minoritized groups, thus also reducing the risk of tokenism.

### Unique risks and benefits

Cancer ED presents unique risks and benefits for participants. In routine delivery of early cancer detection services false positives and false negative test findings, each with its repercussions; overdiagnosis and overtreatment of slow-growing or indolent cancers; and detection of early cancers for which a treatment has not yet proven effective are known risks [42–45]. These risks are also present in ED cancer research settings, varying in extent depending on the duration of the study. Still, uncertainty is a structural component of ED trials and research, especially when studies involve biomarker development [46]. Consequently, researchers may be initially unable to assess all the risks and benefits for participants [46–48]. On the other hand, participation in cancer ED studies also offers specific benefits equally applicable to research and care settings: participants may receive an earlier diagnosis or learn about their chances of developing cancer in the future and be monitored accordingly [13, 42, 47, 49]. Results may also compel lifestyle changes that reduce cancer risks.

Fieldwork research demonstrated that trust between research teams and minoritized groups can be damaged by mismatched expectations, especially when researchers do not clearly communicate the risks or benefits of participation [36, 46, 50–52]. Research teams foster trust when they transparently communicate these issues and make rigorous plans for follow-up care (see recommendation #9) [46]. Communication is especially important when research studies use tests that have yet to be clinically validated, or when positive results cannot be relayed back to participants. Clarity and accessibility are essential when communicating these risks and benefits, ensuring participants can ask questions and voice concerns in an accessible way [46, 51, 52]. Ideally, this means face-to-face communication, in a language both parties speak fluently, with written or verbal information to access later (see recommendation #3) [21, 37, 53]. Moreover, a clinical environment may trigger negative perceptions for research participants based on previous suboptimal healthcare experiences [54, 55]. It is therefore important to provide a non-clinical, neutral and convenient space for these conversations to create a distinction between healthcare services and research that is clear to all participants [21, 56].

### Compensation and support

For cancer ED research to be equally accessible to everyone, it is crucial that any expenses incurred by the participants be covered by research teams [4, 57]. Moreover, fieldwork participants noted that providing resources such as access to the internet, child-caring services, and disability aids might help overcome some of the practical barriers to participation in research that minoritized groups face. Beyond practical reasons, compensating participants demonstrates that research teams value the time and effort involved in participation [58–62]. In addition to compensating individual research participants, community groups and organisations also need to be compensated for their time and work in research projects [60]. Workshop participants noted the difficulties in reimbursing community groups that are not listed as institutional suppliers which can lead to significant delays in payment that damage relationships [63]. Developing clear and accessible processes at research institutions for compensating community groups will likely support connections with organisations that fall outside traditional forms of public involvement, diversifying research inputs [60, 63].

### Representative samples

To understand how an intervention impacts people from various ethnicities and socio-economic backgrounds, participants need to be representative of the population it seeks to serve [11, 21, 37, 64–69]. Research also shows that published biomedical studies often lack crucial information about the use of culturally sensitive recruitment methods [21, 30, 70]. By documenting how trials engage and recruit participants, particularly from groups underrepresented in cancer ED research, workshop participants recommended that researchers could learn from each other and avoid repeating mistakes [71, 72].

### Participant demographic data

Historically, the collection of data regarding minoritized communities in medical research has been inadequate [73, 74]. Currently, the majority of clinical trials do not document ethnicity data [21, 37, 69, 75]^3^. People with completely different ethnicities are lumped together in broad categories such as ‘Black’, ‘White’, or ‘Other’ [66]. Consequently, knowledge about disparities in cancer incidence and outcomes, as well as the effectiveness of particular tests or treatments between different groups is limited [73, 76]. For this reason, appropriately recording participants’ demographic data has been suggested as standard practice in clinical research studies [11, 67]. This includes data on; the medical condition in question, sex and gender, age, ethnic group, geography and the overall representativeness of the trial, including how well the study population aligns with the target population [11, 67]. Workshop participants also recommended adding sexual orientation, disability, and religion to the above characteristics, as well as collecting data on postcodes rather than broader ‘geography’ when relevant. However, fieldwork participants cautioned research institutions to be mindful of communities’ concerns regarding data usage and sharing: Assurance of privacy, confidentiality (which can be formalised through a certificate of confidentiality^4^), and the option to not answer delicate questions is essential to improve trustworthiness in research [77–80]. Especially when collecting data for minoritized groups that have been negatively impacted by researchers’ misuse of data in the past [74].

While data protection laws provide important and necessary safeguards by mandating anonymisation and aggregation [81], detailed data are essential to expose disparities, uncover underlying social determinants of health and identify specific population needs [73]. These data help identify how the research in question may differentially impact participants from various ethnicities, socio-economic backgrounds, and abilities, and intersections of those characteristics [11, 37, 68, 82].

### Post-research support

In several research contexts, if the study’s intervention demonstrates to be beneficial, ethical and funding guidelines mandate the intervention to be offered to any control group that did not receive it during the study. However, depending on the research design and stage, cancer ED studies may not be able to provide participants with results, though transparency towards participants should always be prioritised [83]. Aware of that possibility, fieldwork participants explained that if giving individual test results is not an option in the study, this should be explained clearly from the outset to manage participants’ expectations. Alternatively, if a test is validated, or further testing is possible, a funded follow-up plan, created in collaboration with participant representatives about whether, how, and when to communicate a positive result and the steps that can be taken to ensure participants’ wellbeing demonstrates that researchers care about participants [30, 84, 85]. The perception that medical insurers are withholding services such as follow-up care from participants can yield strong reactions of distrust [44]. Moreover, in countries where healthcare and health insurance are not guaranteed, funding institutions and research teams need to devise a strategy to cover the costs of follow-up care when needed. Findings from the literature review reveal that by offering follow-up care and addressing the psychosocial impact of research participation, fear of a cancer diagnosis can be better handled [46]. This might encourage socially and financially vulnerable people, who may be at a greater disadvantage to deal with the impacts of such research results, to take part in ED cancer research.

### Sharing results

Fieldwork participants noted that when study results are not fed back, it can damage trust between participants from minoritized groups and researchers, seeding doubts regarding the real purpose and benefit of the clinical study [21]. Participants also expressed they would be less likely to enrol in a study that would not provide planned dates for disseminating results. They wanted to know how they contributed to the research and how those results can effect change in their communities. Feedback of results is crucial to addressing the concerns members of minoritized groups often have that ‘nothing in research is for them’ [24].

Members of some minoritized groups reported during fieldwork that the anxiety caused by a long wait for results would cause them not to participate at all, even if the study was to offer them direct benefits such as potentially detecting their cancer early [47]. This is especially relevant in the context of cancer ED, where trial participants are usually cancer-free to the best of their knowledge [47]. Research teams are advised to consider the timeliness and inclusivity in which results are communicated and participation is acknowledged [24, 30]. This is crucial for trust-building, minimising stress and providing reassurance to participants [24, 30, 46]. Thoughtful acknowledgement of participants’ time, effort and commitment might help them reflect on their research experience as positive and meaningful, increasing the chances they or their social networks will participate in ED cancer research in the future [24].

### Research training

Insights from the literature and fieldwork highlight that harm can be caused when researchers come with set ideas, respond insensitively to lived experiences, or wrongly assume that they know how to engage minoritized groups in research because they have worked with the public in other clinical contexts [15, 16, 86]. One way of mitigating such harms is through cultural humility training^5^ for research teams, which has been proven to improve research participation amongst minoritized groups [21, 37, 70, 89–92]. Published recommendations on including underserved groups in biomedical research advise that researcher training could address: the significance of including minoritized groups in research, group-specific barriers to participation, cross-cultural communication and dispelling harmful biases and stereotypes that researchers may have [21, 37]. Among workshop participants, there was consensus that cultural competency training should be mandated by funders and research institutions and developed in collaboration with appropriate community organisations [21]. Moreover, to foster and sustain mutually beneficial relationships with minoritized groups in ED cancer research, workshop participants suggested further training on health disparities among minoritized groups; best practices in community engagement approaches; and responding to (historical) experiences of marginalisation, trauma, ableism and racism in research [65].

### Diversifying research teams

Evidence suggests that diversifying research teams increases the participation rates of minoritized groups and improves data collection on sociodemographics crucial to addressing health inequalities in research [11, 90, 93–95] (See recommendation 8). Research institutions and funders have the potential to play a pivotal role in ensuring the diversification of the future research workforce [11, 96]. They can support the progression pathways of people from minoritized groups in ED cancer research by organising outreach and mentorship initiatives, creating equitable employment opportunities, ensuring an inclusive working environment and incorporating the diversity of research teams into funding considerations [97–99]. For the design and execution of ED cancer research to truly represent the priorities and perspectives of minoritized groups, research teams need to reflect the diversity of the communities they work with. To avoid tokenism, institutions and funders are encouraged to set measurable goals and collaborate with representatives of minoritized groups in developing strategies to diversify the research force at all levels, including senior positions [99]. Anything less, risks reinstating hierarchies which played an active role in people’s minoritization in the first place, perpetuating an “us” (researchers) vs. “them” (minoritized groups) polarising logic to the researcher-participant relationship.

## Discussion

Past recommendations and guidance on improving the participation of underrepresented groups in medical research have emphasised the importance of many recommendations presented here [4, 7, 11, 13, 21, 29, 30, 37, 58, 68, 70–73, 90–92, 100]. Yet REPRESENT research activities centred on the context of cancer ED research, offering some unique insights: Recommendation #2 focuses on institutionalising processes to share resources with minoritized groups beyond individual research projects. This finding emerged from the Manchester and Oregon fieldwork case studies and was further echoed by workshop participants who saw it as a necessary condition to move towards a long-term culture change in ED cancer research. Furthermore, recommendations #5 and #9 were formulated to address dilemmas that are specific to the context of ED cancer research, such as ensuring follow-up care when cancer is detected for minoritized participants and attending to the unique risks of cancer ED studies such as overdiagnosis and false positives. Our recommendations are not a magic bullet for these complex dilemmas, which require systemic, multi-sectoral, and multilevel solutions more far-reaching than the scope of research institutions, yet they intend to improve the trust of minoritized groups in cancer ED research. To further confirm this, pilot studies providing empirical evidence to test this would be needed.

Our twelve recommendations have several strengths. First, they have been developed with a wide range of relevant stakeholders in the US and UK, increasing their acceptability and uptake [101]. Workshop participants emphasised that past roadmaps have not allocated responsibility to specific stakeholder groups, resulting in recommendations not being enforced or implemented. For this reason, the recommendations in this paper detail the specific role of each contributing group; funders, institutions and research teams (see Table 3). Moreover, discussions were informed by a range of research methods including qualitative fieldwork and a scoping literature review, combining the strengths of triangulating diverse data collection and analysis methods. This approach enabled the REPRESENT team to address context-based and real-life dilemmas identified in the field, using a multistakeholder approach to then produce actionable findings.

One potential limitation is that while invitations were extended to a wide range of stakeholders, 22 out of 33 participants of the workshop were researchers (9 from the REPRESENT team), replicating existing relations of underrepresentation in research. This was due, in part, to the nature of the workshop. Asking people to commit to three consecutive mornings or evenings posed a challenge to many potential participants who, although showing interest in our work, could not make themselves available for that long, being burdened with their day-to-day responsibilities. Professional development credits or other forms of compensation may encourage the future attendance of engagement practitioners. Public contributors were offered Zoom training, monetary compensation and childcare funds, but those measures did not assuage the feeling that many public contributors expressed when doubting their abilities and knowledge to meaningfully contribute to the exercise. This issue reflects the nature of working with minoritized groups in the first place, where sparsity in research participation over time engenders fewer opportunities and role models to learn from. Hence, more stakeholders who are public contributors, engagement practitioners and funders would have been desirable to inform consensus in a more inclusive and representative manner.

Because there is much overlap between barriers to participation in cancer ED research and other biomedical fields, and a lack of trust between the public and researchers is a known problem in clinical research and care [102–104], outputs from the REPRESENT study may be considered transferable to other areas of clinical research. Moreover, the twelve recommendations presented here will provide insights and actionable suggestions for countries in which cancer ED ranks highly on the national research agenda. Existing roadmaps and recommendations for improving participation in cancer research have focused largely on increasing enrolment in clinical trials [21, 37, 91, 92]. The recommendations presented here aim to foster participation of minoritized groups in other kinds of research, such as; surveys, cohort studies, and qualitative research, which are also crucial to improving cancer ED outcomes and care [105–108].

These consensus-based recommendations are informed by empirical evidence. Nevertheless, this study has not attempted to test them. One fruitful area for future research on the underrepresentation of minoritized groups in ED cancer research may be trialling the implementation of the recommendations outlined above, testing their efficacy and limitations. Deploying these recommendations with specific minoritized groups could help tailor approaches to particular settings, contexts and conditions, providing researchers with nuanced guidance on what works, for whom and under what circumstances [109–111]. While doing this, knowledge transfer is essential according to workshop participants: Widely sharing insights gained about involvement and recruitment of minoritized groups in cancer (ED) research with other organisations and research teams is needed to overcome ‘pilotitis’ and sparsity of research in this area. To achieve this, further engagement with funding bodies, public contributors and policymakers in this area is needed.

## Conclusion

The twelve recommendations presented in this manuscript invite research teams to go beyond isolated public involvement and engagement activities and recruitment efforts for cancer ED research. Involving groups that are usually underrepresented in cancer ED research and building trusting relations requires a re-thinking of standard practices that have long created asymmetry in researcher/participant relationships. Detecting cancer early is a joint effort which requires an approach that works for everyone. Rather than research teams ‘extracting’ what they need for the advancement of ‘their’ research, mutually beneficial relationships with relevant minoritized groups can help participants from all walks of life feel invited, safe and inspired to participate in and contribute to cancer ED research. The actions suggested in these recommendations aim to initiate a wider research paradigm change. One in which mutually beneficial, equal, and trusting relationships between researchers and wider society become embedded within research institutions and practice rather than being left up to the goodwill of individuals.

## Appendix

**Methods**

Community engagement approaches developed by OHSU and Vocal in Manchester were studied by the REPRESENT team as part of two comparative case studies. A common assessment criterion was developed to understand how these approaches work, what the success criteria are, and how we can learn from each centre regarding engagement, inclusion and training in cultural competency. Using an interpretative analysis framework [112], the assessment framework paid particular attention to the mechanisms and enablers in those contexts that increase the representation of underserved groups in ED cancer research, promoted trust-building among groups usually alienated by biomedical interventions, and engendered long-term changes in the communities. These case studies aimed to explore the qualitative impact that OHSU and Manchester approaches have on scientists, practitioners and members of underserved communities, potentially changing power dynamics in the research process and satisfying communities’ research needs [28].

**Fieldwork in Oregon**

Fieldwork was conducted from March to April 2022 with the Community Outreach, Research and Engagement (CORE) team at the Oregon Health & Science University (OHSU) in Oregon, US. The CORE team aim to build partnerships between researchers and communities that address community-driven health priorities by offering a range of research engagement, outreach, and training initiatives [113]. The Oregon case study focused on two evidence-based research engagement models conducted in Spanish that were carried out by the CORE team: Community Engagement Studios (CES), that is, structured conversations in which community stakeholders provide researchers with feedback (*n* = 2) [114]; and a Community Readiness Assessment Model (CRAM), a peer-to-peer interview process scored via a Likert scale (1–5) to assess the readiness of a group to take action (*n* = 1) on particular issues [115]. First, the REPRESENT team observed two online CES’ on ED cancer research. Each included ten Hispanic and Latino community experts from Central Oregon (*n* = 10, *n* = 10) and was facilitated in Spanish. They centred on two questions: 1) What methods help us increase participation of underrepresented communities in early cancer detection research? And 2) How can we build trust between researchers and underrepresented communities within early cancer detection research? Then, REPRESENT team members observed a CRAM carried out with sixteen Latino and Hispanic community stakeholders from Corvallis, Oregon, who served as both interviewers (*n* = 4) and interviewees (*n* = 12). The CRAM addressed the question: How can we create context-specific community engagement models to build trust and social acceptability of early cancer detection research across groups traditionally underrepresented in clinical cancer research?

**Fieldwork in Manchester**

Fieldwork was carried out with Vocal, a non-profit organisation hosted by the Manchester University NHS Foundation Trust [116] for 4 weeks in May 2022. Vocal specialise in organising and facilitating Patient and Public Involvement and Engagement (PPIE) activities to include people from diverse backgrounds in health research. Vocal carried out four community workshops either in English or with live translation from other languages. Questions were centred on trust and participation in cancer ED research. Each workshop was held with and tailored to public contributors from the following minoritized groups in Manchester: African Caribbean heritage (*n* = 7), South Asian heritage (*n* = 9), East Asian heritage (*n* = 9) and people living in economically deprived neighbourhoods (*n* = 9). The workshop questions were: 1) How can we include all communities in early detection cancer research? And 2) How can we build trust between researchers and communities? The questions were co-designed with REPRESENT team members, public engagement practitioners, and BRAG (Vocal’s Black Asian and Minority Ethnic Research Advisory Group), and due to the multi-stakeholder input, they were also chosen as the guiding questions of the REPRESENT workshop.

## Footnotes

1 Minoritized” has been recommended as opposed to minorities to reflect that individuals do not merely become statistical minorities but have been actively minoritized by dominant groups. Moreover, whilst certain ethnic/racial groups may be a minority in the US or the UK, they may be majorities globally speaking [3].

2 See Appendix-1 Methods

3 For example, less than 33% of cancer trials in the US were found to report on race and ethnicity data [37].

4 For more information, see N IH (2021).

5 A more reflexive form of the well-established term cultural competency, whereby researchers develop an awareness of their own bias and limitation of their knowledge [87, 88].
